# Health facility management and primary health care performance in Uganda

**DOI:** 10.1186/s12913-022-07674-3

**Published:** 2022-03-01

**Authors:** June-Ho Kim, Griffith A. Bell, Asaf Bitton, Eesha V. Desai, Lisa R. Hirschhorn, Fredrick Makumbi, Elizabeth Nabiwemba, Hannah L. Ratcliffe, Fred Wabwire-Mangen, Simon P. S. Kibira, Dan Schwarz

**Affiliations:** 1 Ariadne Labs (Harvard T.H. Chan School of Public Health & Brigham and Women’s Hospital), 401 Park Drive, 3rd Floor East, Boston, MA 02215 USA; 2 Division of General Medicine and Primary Care, Brigham and Women’s Hospital, MA Boston, USA; 3 Northwestern University Feinberg School of Medicine, Chicago, IL USA; 4 Makerere University School of Public Health, Kampala, Uganda; 5 Division of Global Health Equity, Brigham and Women’s Hospital, Boston, MA USA

**Keywords:** Management, Health facilities, Primary health care, Quality, Essential drugs

## Abstract

**Background:**

Primary health care is a critical foundation of high-quality health systems. Health facility management has been studied in high-income countries, but there are significant measurement gaps about facility management and primary health care performance in low and middle-income countries. A primary health care facility management evaluation tool (PRIME-Tool) was initially piloted in Ghana where better facility management was associated with higher performance on select primary health care outcomes such as essential drug availability, trust in providers, ease of following a provider’s advice, and overall patient-reported quality rating. In this study, we sought to understand health facility management within Uganda's decentralized primary health care system.

**Methods:**

We administered and analyzed a cross-sectional household and health facility survey conducted in Uganda in 2019, assessing facility management using the PRIME-Tool.

**Results:**

Better facility management was associated with better essential drug availability but not better performance on measures of stocking equipment. Facilities with better PRIME-Tool management scores trended towards better performance on a number of experiential quality measures. We found significant disparities in the management performance of primary health care facilities. In particular, patients with greater wealth and education and those living in urban areas sought care at facilities that performed better on management. Private facilities and hospitals performed better on the management index than public facilities and health centers and clinics.

**Conclusions:**

These results suggest that investments in stronger facility management in Uganda may strengthen key aspects of facility readiness such as essential drug availability and potentially could affect experiential quality of care. Nevertheless, the stark disparities demonstrate that Uganda policymakers need to target investments strategically in order to improve primary health care equitably across socioeconomic status and geography. Moreover, other low and middle-income countries may benefit from the use of the PRIME-Tool to rapidly assess facility management with the goal of understanding and improving primary health care performance.

## Background

The 2018 Astana Declaration revitalized the global drive to strengthen primary health care (PHC) as a way to improve the responsiveness and readiness of a country’s health care system [1]. In light of the 2020 COVID-19 pandemic, it has become clear that PHC must be strengthened in order to effectively deliver accessible, coordinated, comprehensive, and high-quality health care even during a crisis [2].

In high-income countries, facility management has been shown to be a necessary component of high quality PHC in order to optimize the effectiveness and efficiency of many health services [3–5]. Conversely, though there has been increased interest in measuring facility management performance and structures in low and middle-income countries (LMICs), a large measurement gap persists [6–10].

A new rapid-turnaround and nationally representative survey methodology was developed to assess the management of facilities through the Performance Monitoring for Action (PMA) initiative and implemented in Ghana in 2016 and 2017. Within this survey, the PRImary care facility Management Evaluation tool (PRIME-Tool) was designed to measure facility management in LMICs [7, 11]. In Ghana, the PRIME-Tool demonstrated that better facility management was associated with better stocking of essential drugs, higher trust between the patient and provider, better ease of following a provider’s advice, and better patient-reported quality of care [7].

Uganda is a low-income country with a fast growing population–between 1980 and 2015, the population tripled in size–and an increasing double burden of communicable and non-communicable diseases [12]. PHC is predominantly delivered through government-run public facilities, which have a national and regional referral network and account for 66% of the country’s health service delivery outputs [12]. Uganda provides PHC through its National Minimum Health Care Package, which aims to provide equitable health promotion, disease prevention, and child and maternal health through providing access to a list of essential services to the entire population [13]. The decentralized health system is divided into county, sub-county, parish, and village levels. Medical doctors are most often found in urban areas with fewer found in rural areas.

In its drive towards universal health coverage, several components of the PHC system in Uganda have required improvement. In 2017, Ugandan doctors staged a major strike out of protest about “low wages, drug shortages, and lack of equipment” [14]. Some of these issues may be best addressed at a national level, such as wage increases. However, there is significant local variability for certain factors, which suggests opportunity for facility-level improvement. For example, for-profit facilities have been found to have 98% higher availability of essential medicines for treating non-communicable diseases, compared to public facilities [15]. Referral and general hospitals also had approximately 100% higher counts of essential medicine compared to primary health centers. There were also geographic disparities with facilities in the Kampala capital region having better drug availability than those in the North and East [15]. This heterogeneity may suggest variations and disparities in facility management and quality that may have important implications for Ugandan policymakers.

In this study, we build on previous work in Ghana to utilize the PRIME-Tool and evaluate facility management in Uganda in order to understand its association with PHC delivery readiness and quality. We examined variations in PRIME-Tool management scores across facility types and regions as well as the associations of management performance with facility-level outcomes and experiential quality of care. From prior studies, there are known health disparities across Uganda, and other countries like Ghana have demonstrated that better facility management may be associated with positive PHC outcomes. Therefore, we hypothesized better facility management in Uganda would be associated with higher performance on measures of readiness (essential drug and equipment availability) and experiential quality. However, we predicted that better managed facilities would be more likely to be accessed by wealthier, better educated, and urban resident Ugandans and that these facilities would be more likely to be larger, privately managed, and providing a higher level of care compared to facilities that were more poorly managed.

## Materials and methods

### Survey design and administration

We developed, administered, and analyzed a cross-sectional household and health facility survey conducted in Uganda in 2019 by the Performance Monitoring for Action (PMA) project (www.pmadata.org) implemented by the authors’ institute. The household survey of patient experience was nationally representative and developed based on measures of responsiveness from the WHO World Health Survey Responsiveness Module [16] while the facility management portion of the survey, or the PRImary care facility Management Evaluation tool (PRIME-Tool), was adapted from the World Management Survey as previously described [7].

Trained resident enumerators collected data from 110 enumeration areas from 10 statistical subregions generated by the Uganda Bureau of Statistics, with probability proportional to size using a National Census master sampling frame stratified by urban–rural areas. The team surveyed 4,823 randomly selected households about their demographics, care-seeking behaviors, self-reported health status, and experiences with the Ugandan health care system. All members of the household aged 15 + were surveyed, but only one individual was randomly selected to complete the primary health care module. Unlike the previous sample population from the survey conducted in Ghana, which interviewed only women of reproductive age, we surveyed a nationally representative sample of both women and men as well as older individuals in Uganda.

For the health care facility surveys, a sample of 393 public and private health care facilities that serve the 110 enumeration areas were surveyed. At each health facility, survey enumerators asked to speak with the head of the facility, which included the Medical Director/Superintendent, Director of Nursing, or Facility In-charge at hospitals and Nurse, Midwife, or Physician Assistant at health centers. The facility surveys were administered in English, while the household surveys were in either English or an appropriate local language. The questionnaires were translated and back translated prior to the interviews. Data were collected from March to May 2019. All methods were performed in accordance with the relevant guidelines and regulations.

### Study variables

For the PRIME-Tool management score, we assigned 27 indicators from the facility survey into five predefined management domains (target setting, operations, human resources, monitoring and evaluation, and community engagement) based on the World Management Survey as previously described [7]. Indicators were rescaled from 0 (lowest possible score) to 1 (highest possible score) while “do not know” and missing responses were assigned a value of zero when deemed that the facility respondent should know the answer. Scores for each of the five domains were determined by averaging the indicators within each domain. Thereafter, the scores across the five domains were averaged to produce a summary facility management score for each health facility.

Based on previous work on experiential quality [17], the patient-level outcomes of interest were:

1. Rating of waiting times for before seen at the facility
2. Rating of facility cleanliness
3. Rating of trust in the skills and abilities of the health workers at the facility
4. Rating of the level of respect shown by the provider
5. Rating of the provider’s ability to explain information in an understandable way
6. Rating of ease or difficulty in following the provider’s advice
7. Likelihood of returning or bringing the patient’s children to the facility for future health care
8. Overall rating of quality of care

Patient-level outcomes were scored on a 5-point Likert scale and rescaled from 0 to 1.

The facility-level outcomes of interest were identified based on the availability of data within the facility survey and feasibility of analysis. The drug and equipment indices were selected as measures of health system readiness and assessed in a previous use of the same survey in Ghana [7]. These outcomes were defined in the following manner:

1. Essential drug index—the proportion of available drugs at the facility from a prespecified list of drugs deemed vital or essential in the 2016 Essential Medicines and Health Supplies List for Uganda [18] (scaled from 0 to 1)
2. Equipment index—the proportion of available and functional equipment from a list of six basic pieces of equipment (thermometer, stethoscope, sphygmomanometer, weighing scale, sterilization equipment), which were identified from the Service Delivery Indicators lists of essential equipment [19] (scaled from 0 to 1)

For the multivariable models, our covariates of interest were at the facility level and included facility type, region, managing authority (public, private, faith-based organization), and facility size, defined by the number of beds. Patient-level characteristics of interest were age, level of educational attainment, marital status, insurance coverage, urban or rural region of residence, and needing to borrow money or sell something in order to afford the health facility visit.

### Data analysis

Within our analytic sample, all health facilities that provided primary health care services in Uganda were included. Facilities that were surveyed but did not offer primary health services, such as chemists and pharmacies, were excluded. Individuals who sought care from a health facility in our facility sample within the six months prior to the survey were included in the analysis of patient-level outcomes.

We first examined the characteristics of our participants and facilities using descriptive statistics (counts and percentages). We then explored PRIME-Tool management scores across domains and Ugandan geographic regions. In order to examine the distribution of facility management scores, we divided management scores into quintiles and compared the highest quintile to the lowest across facility-level characteristics. We then compared the facility management score to our patient-level outcomes of interest using Poisson regression with robust standard errors. We estimated risk ratios to determine whether patients who received care at facilities in the highest quintile of management score were more likely to report the highest levels of care experience (coded as 1 = ”excellent” or the highest score, and 0 = all other responses) compared to those in the lowest quintile. Finally, to investigate the relationship between essential drug index and equipment index, we used linear regression with robust standard errors to estimate marginal means of each outcome within each quintile of management score. Poisson and linear regression models were fit without adjustment and then adjusted for region, managing authority, and facility type.

Informed consent was obtained from all subjects and for subjects under 18 and for non-literate participants, from a parent and/or guardian. The study protocol was approved by the Institutional Review Boards at the authors’ institutes and ultimately the Uganda National Council for Science and Technology (Ref: SS4869). Funding was provided by the Bill & Melinda Gates Foundation, which played no role in the study design; data collection, analysis, and interpretation; manuscript writing; or submission for publication.

## Results

A total of 292 health facilities distributed across 10 sub regions were included in our analysis (Table 1). Of the 292 total health facilities, there were 36 (12.3%) health clinics, 65 (22.3%) health center II, 83 (28.4%) health center III, 59 (20.2%) health center IV, and 49 (16.8%) hospitals. Two-hundred twenty-nine (78.4%) of the health facilities were managed by the government.

**Table 1:** Characteristics of Health Facilities, 2019

	**N**	**%**
**Region**		
Central 1	26	9.1
Central 2	35	12.2
East Central	40	13.9
Eastern	39	13.6
Kampala	26	9.1
Karamoja	17	5.9
North	31	10.8
South-West	33	11.5
West Nile	14	4.9
Western	26	9.1
Total	287	100
**Number of Beds**		
0–36	36	34.6
40–100	38	36.5
103–400	30	28.8
Total	104	100
**Type of Facility**		
Hospital	49	16.8
Health Center IV	59	20.2
Health Center III	83	28.4
Health Center II	65	22.3
Health Clinic	36	12.3
Total	292	100
**Managing Authority**		
Government	229	78.4
NGO	3	1
Faith-based Organization	23	7.9
Private	37	12.7
Total	292	100

A total of 1,339 Ugandans who reported seeking care in the prior six months were included in the patient experience analysis (Table 2), including 920 women (68.7%) and 419 men (31.3%). The largest proportion of respondents (28.1%) were between the ages of 25 to 34 years old and 72.6% of the sample were distributed within the lowest three wealth quintiles in Uganda. Over half (59.4%) of respondents reported having only completed primary school. 86.6% of the sample population lived in rural areas.

**Table 2:** Demographics of Survey Participants, 2019

	**N**	**%**
**Age Category**		
15–19	173	12.9
20–24	208	15.6
25–34	376	28.1
35–44	262	19.6
45–54	149	11.1
55–64	93	7
65 +	77	5.8
Total	1338	100
**Sex of Household Member**		
Male	419	31.3
Female	920	68.7
Total	1339	100
**Quintile of Wealth**^**a**^		
Quintile 1	371	27.7
Quintile 2	328	24.5
Quintile 3	272	20.4
Quintile 4	247	18.5
Quintile 5	120	9
Total	1338	100
**Highest Level of School Attended**		
Missing	1	0.1
Never	217	16.2
Primary	795	59.4
O	238	17.7
A	22	1.6
Tertiary	53	3.9
University	13	1
Total	1339	100
**Marital Status**		
Currently Married	487	36.4
Currently Living with Partner	438	32.7
Divorced	137	10.3
Widowed	96	7.2
Never Married	180	13.5
Total	1338	100
**Neighborhood**		
Urban	179	13.4
Rural	1160	86.6
Total	1339	100
**Services Covered by Insurance**		
Missing	1	0.1
No	1332	99.5
Yes	5	0.4
Total	1338	100

^a^Quintile 1: lowest quintile; Quintile 5: highest quintile

PRIME-Tool management scores varied across domains, regions, and types of facilities (Table 3). Across all facilities, the median score (on a scale of 0 to 1) was highest for human resources (median 0.83, IQR 0.42) and monitoring and evaluation (median 0.83, IQR 0.29). Operations had the lowest overall score (median 0.50, IQR 0.33). The Kampala region performed the poorest across the domains with median scores ranging from 0.15 to 0.75, while the Western region scored highly across several domains, ranging from 0.33 to 1.00. Across the domains, hospitals and health center IV facilities generally received higher scores compared to the lower-level health centers and clinics.

**Table 3:** PRIME-Tool management score domains by region and facility type

	**Target setting**		**Operations**		**Human resources**		**Monitoring and evaluation**		**Community engagement**	
	median	IQR	median	IQR	median	IQR	median	IQR	median	IQR
Total (*n* = 287)	0.667	0.667	0.500	0.333	0.833	0.418	0.833	0.291	0.700	0.400
**Region**										
Central 2 (*n* = 35)	0.667	0.667	0.500	0.500	0.833	0.250	0.864	0.375	0.700	0.400
Central 1 (*n* = 26)	0.333	0.667	0.458	0.333	0.750	0.333	0.849	0.666	0.550	0.600
East Central (*n* = 40)	0.667	0.167	0.500	0.417	0.750	0.500	0.718	0.239	0.700	0.375
Eastern (*n* = 39)	1.000	0.333	0.500	0.333	0.833	0.333	0.895	0.115	0.850	0.200
Kampala (*n* = 26)	0.333	0.667	0.333	0.333	0.750	0.750	0.396	0.573	0.150	0.500
Karamoja (*n* = 17)	0.667	0.667	0.500	0.167	0.833	0.333	0.823	0.125	0.550	0.200
North (*n* = 31)	0.667	0.667	0.500	0.500	0.833	0.250	0.864	0.156	0.650	0.200
South-West (*n* = 33)	0.667	0.667	0.500	0.333	1.000	0.250	0.823	0.291	0.600	0.400
West Nile (*n* = 14)	0.667	0.333	0.500	0.333	1.000	0.168	0.958	0.094	0.700	0.050
Western (*n* = 26)	0.667	0.333	0.333	0.333	1.000	0.250	0.869	0.178	0.725	0.450
**Facility type**										
Hospital (*n* = 48)	1.000	0.333	0.667	0.167	1.000	0.168	0.921	0.094	0.750	0.300
Health Center IV (*n* = 58)	0.667	0.333	0.667	0.167	0.915	0.250	0.885	0.115	0.750	0.250
Health Center III (*n* = 82)	0.667	0.333	0.500	0.167	0.915	0.250	0.864	0.178	0.725	0.350
Health Center II (*n* = 63)	0.667	0.333	0.167	0.333	0.750	0.415	0.698	0.271	0.600	0.250
Health Clinic (*n* = 36)	0.000	0.333	0.333	0.208	0.250	0.625	0.234	0.307	0.100	0.150

Table 4 shows the differences in characteristics among patients who sought care at facilities with the highest and lowest PRIME-Tool management score quintiles. There are substantial differences in education levels between patient groups, with a higher proportion of patients with education beyond the primary level attending better-managed facilities. Of the population that sought care at the lowest management quintile facilities, less than 19% had an education higher than primary school. Meanwhile, of facilities in the highest quintile of the management score, nearly 30% were educated beyond primary school. Fewer respondents (39.7%) who attended the lower management facilities reported borrowing money or selling possessions to afford care compared to those seeking care at highest management quintile facilities (55.4%). Very low rates of health insurance were reported regardless of management quintile (0% versus 2.4% at the lowest and highest quintile facilities respectively), and there were no substantial differences in gender.

**Table 4:** Patient characteristics across the highest and lowest PRIME-Tool management score quintiles

	Lowest quintile		Highest quintile		Total	
	No	%	No	%	No	%
**Age**						
15–19	33	13.3	23	8.5	173	12.9
20–24	46	18.9	42	15.8	208	15.6
25–34	63	25.6	69	26.1	376	28.1
35–44	46	19	48	17.9	262	19.6
45–54	29	11.9	44	16.7	149	11.1
55–64	11	4.4	25	9.5	93	7
65 +	17	6.9	15	5.5	77	5.8
Total	245	100	266	100	1339	100
**Sex of household member**						
Male	81	32.9	94	35.3	419	31.3
Female	164	67.1	172	64.7	920	68.7
Total	245	100	266	100	1339	100
**Quintile of wealth**^**a**^						
Quintile 1	37	15.1	50	18.6	371	27.7
Quintile 2	85	34.8	56	21.1	328	24.5
Quintile 3	74	30.4	74	27.7	272	20.4
Quintile 4	32	12.9	60	22.5	247	18.5
Quintile 5	17	6.8	27	10	120	9
Total	245	100	266	100	1339	100
**Highest level of school attended**						
Never	47	19.3	36	13.5	217	16.2
Primary	152	62.1	151	56.7	795	59.4
O	35	14.4	56	21.1	238	17.8
A	4	1.5	5	2	22	1.6
Tertiary	5	2	12	4.5	53	3.9
University	2	0.8	6	2.2	13	1
Total	245	100	266	100	1338	100
**Marital Status**						
Currently Married	75	30.5	84	31.4	487	36.4
Currently Living with Partner	98	39.9	100	37.4	438	32.7
Divorced	20	8.1	33	12.4	137	10.3
Widow	13	5.3	20	7.4	96	7.2
Never Married	40	16.1	31	11.5	180	13.5
Total	245	100	266	100	1339	100
**Neighborhood**						
Urban	15	6	30	11.3	179	13.4
Rural	230	94	236	88.7	1160	86.6
Total	245	100	266	100	1339	100
**Region**						
Central	79	32.3	45	16.7	314	23.4
Eastern	43	17.4	95	35.8	323	24.2
Northern	30	12.1	29	10.8	335	25
Western	94	38.2	98	36.7	367	27.4
Total	245	100	266	100	1339	100
**Services Covered by Insurance**						
No	245	100	263	98.6	1332	99.6
Yes	0	0	4	1.4	5	0.4
Total	245	100	266	100	1338	100
**Managing Authority**						
Government	229	93.3	221	82.9	1253	93.6
NGO	3	1.4	0	0	3	0.3
Faith-based Organization	0	0	45	17.1	70	5.2
Private	13	5.3	0	0	13	1
Total	245	100	266	100	1339	100
**Type of Facility**						
Hospital	17	6.8	134	50.3	327	24.4
Health Center IV	23	9.4	90	33.9	275	20.5
Health Center III	56	22.8	28	10.3	449	33.6
Health Center II	136	55.7	15	5.5	275	20.6
Health Clinic	13	5.3	0	0	13	1
Total	245	100	266	100	1339	100
**Has any insurance or is a member of a mutual health**						
No	245	100	260	97.6	1328	99.2
Yes	0	0	6	2.4	10	0.8
Total	245	100	266	100	1338	100
**Borrowed money or sold something to afford the costs of care/treatment**						
No	36	60.3	71	44.6	257	49.8
Yes	24	39.7	89	55.4	259	50.2
Total	60	100	160	100	516	100

^a^Quintile 1: lowest quintile; Quintile 5: highest quintile

At the facility level, there were notable differences in the PRIME-Tool management scores by urban–rural location, public–private status, and type of facility. More rural facilities (94%) made up the lowest management quintile compared to the highest management quintile (88.7%). Similarly, there were more public health facilities, managed by the government, in the lowest management quintile (93.3%) than in the highest management quintile (82.9%). Hospitals were 50.3% of facilities the highest management quintile while comprising 6.8% of the lowest management quintile. Meanwhile, health clinics made up 5.3% of the lowest management quintile while none were in the highest management quintile.

In an analysis adjusted for differences in baseline facility-level characteristics, there were no statistically significant differences in various measures of experiential quality regardless of high or low performance on the PRIME-Tool management score (Table 5). Though the differences were not statistically significant, patients who went to facilities in the highest quintile of management scores provided better ratings of perceived wait times, cleanliness, trust in their providers, perceived ability of providers to explain medical information, perceived ease of following a provider’s advice, likelihood of returning to the facility, and overall patient experience scores.

**Table 5:** Relative risk ratios of achieving the top score of experiential quality measures

	**Unadjusted**			**Adjusted**		
	Relative risk ratio^a^	CI 95%	*P*-value	Relative risk ratio^a^	CI 95%	*P*-value
**The length of wait time at the facility before you were seen?**	1.36	0.42, 4.38	0.6	1.49	0.39, 5.65	0.56
**The cleanliness in the health facility?**	2.2	0.83, 5.82	0.11	2.4	0.97, 5.96	0.058
**How much do you trust the skills and abilities of the health workers at this facility?**	1.3	0.83, 2.01	0.25	1.26	0.83, 1.91	0.27
**The level of respect the provider showed you?**	0.72	0.26, 2.00	0.53	0.66	0.23, 1.93	0.45
**The provider’s ability to explain things in a way that you could understand?**	1	0.42, 2.36	1	1.32	0.47, 3.68	0.59
**How easy or difficult was it for you to follow the provider’s advice?**	1.07	0.66, 1.73	0.79	1.14	0.72, 1.82	0.57
**How likely are you to return or bring your children to this facility for health care in the future?**	1.11	0.79, 1.56	0.55	1.22	0.86, 1.73	0.27
**Overall, taking everything into account, how would you rate the quality of care you received at this facility?**	1.27	0.32, 5.13	0.73	1.25	0.35, 4.47	0.73

^a^Relative risk measured between the 80^th^ and 20^th^ percentiles of the PRIME-Tool management score

Adjusted for baseline facility-level characteristics, Ugandan health facilities in the highest management quintile had essential drug index scores of 0.75 (95%CI [0.70, 0.79]) compared to 0.67 (95%CI [0.60,0.74]) in the lowest management quintile (*p*-value < 0.001) (Table 6). In an unadjusted model, there was a statistically significant trend for performance on the equipment index with the highest PRIME-Tool management score quintile scoring 1.0 (95% CI [0.99, 1.00]) and the lowest quintile scoring 0.82 (95% CI [0.76, 0.87]). However, after adjustment for facility characteristics, the trend was not statistically different (*p* = 0.098).

**Table 6:** Essential drug index and equipment index scores across quintiles of the management index

	**Unadjusted Models**				**Adjusted Models**			
**Essential drug index (EDI)**	EDI score	Lower 95% CI	Upper 95% CI	*p*-value trend	EDI score	Lower 95% CI	Upper 95% CI	*p*-value trend
Quintile 1	0.67	0.59	0.74	< 0.001	0.67	0.60	0.74	< 0.001
Quintile 2	0.62	0.56	0.67		0.69	0.65	0.74	
Quintile 3	0.74	0.69	0.78		0.74	0.70	0.78	
Quintile 4	0.74	0.69	0.79		0.72	0.67	0.77	
Quintile 5	0.81	0.77	0.85		0.75	0.70	0.79	
**Equipment index (EI)**	EI score	Lower 95% CI	Upper 95% CI	*p*-value trend	EI score	Lower 95% CI	Upper 95% CI	*p*-value trend
Quintile 1	0.82	0.76	0.87	< 0.001	0.81	0.73	0.89	0.098
Quintile 2	0.89	0.84	0.94		0.93	0.89	0.97	
Quintile 3	0.96	0.93	0.98		0.95	0.92	0.97	
Quintile 4	0.96	0.94	0.99		0.96	0.93	0.98	
Quintile 5	1.00	0.99	1.00		0.99	0.96	1.01	

^a^Quintile 1: lowest quintile; Quintile 5: highest quintile

## Discussion

In this study, we found that better performance on facility management was associated with better essential drug availability, a key indicator of health system readiness. Meanwhile, facilities with higher PRIME-Tool management scores performed better on an array of patient experience measures, though differences were not statistically significant in an adjusted analysis. These measures included ratings of perceived wait times, cleanliness, trust in their providers, ability of providers to explain medical information, ease of following a provider’s advice, likelihood of returning to the facility, and overall patient experience scores. Finally, there were many notable disparities in facility management performance across several patient and facility characteristics.

Better-managed facilities had higher essential drug availability even after controlling for baseline facility characteristics. This finding is consistent with several other studies across sub-Saharan Africa that suggest that better management may improve supply availability. Prior studies of drug stock outs in sub-Saharan Africa have shown that pharmaceutical shortages were associated with various aspects of facility management such as human resource constraints and supply management [20, 21]. Additionally, in a study in Ghana utilizing the same management index as this analysis, facilities with PRIME-Tool management scores in the 90^th^ percentile had 22% more essential drugs compared to those at the 10^th^ percentile [7].

Essential drug availability is a major component of health system readiness as well as a major factor of Ugandans’ perception of the quality of care [22]. In fact, in a 2003 study, three different categories of stakeholders in the Ugandan primary health care system (health planners, providers, patients) all saw drug availability as fundamental for the quality of care [23]. The Uganda National Medical Stores manage the national procurement and distribution of drugs to districts across the country, but a recent study of antiretroviral medications showed that Ugandan health facilities often devised a variety of internal and external stock management strategies to address drug stock-outs [24]. Taken together with prior research, our study suggests that investments to improve facility management may enable managers to better handle these challenges by designing proactive supply chain strategies, and thus improving this critical aspect of health system readiness and quality.

On the other hand, after adjusting for covariates, our study did not find a significant association between facility management and an equipment index. Though lack of equipment has often been identified as a major concern in Uganda [14], we speculate that equipment procurement, especially because it is likely lower volume and less frequent, may not benefit as much from proactive management skills as drug availability.

While we did not observe significant differences between management performance and the experiential quality of care, the positive trends may suggest that improving management may improve more than just service readiness (as demonstrated through improved drug availability); it may also be a lever towards improving patient experience of care. Nevertheless, experiential quality is influenced by many factors [25, 26], so improving facility management may be only one component of a multifaceted strategy to improve patient experience.

Lastly, there were notable disparities in the management performance of primary health care facilities in Uganda. A greater proportion of facilities classified as hospitals and health center IV had higher management scores than “lower level” facilities while privately managed facilities performed better on management than public health facilities. Additionally, patients who were educated, wealthier, and urban dwelling were more likely to seek care at well-managed facilities than other types of patients.

Interestingly, people seeking care at better managed facilities were also more likely to report that they had to borrow or sell something in order to afford the visit. Our recent study in Ghana demonstrated that many women bypassed their nearest health facilities and sought care at hospitals and private facilities while also paying more out-of-pocket to access those facilities [27]. It is possible that a similar pattern of care-seeking may be occurring in Uganda in which wealthier individuals seek care at “higher level” health facilities that are better managed and pay additional costs, such as those for transportation, in order to afford the visit.

Generally, these disparities are consistent with prior studies that have shown that patients in LMICs with greater wealth and education have better access to health facilities that have better supplies [28]. As previously discussed, for-profit facilities in Uganda had 98% higher availability of essential medicines for treating non-communicable diseases, compared to public facilities [15] while referral and general hospitals had approximately 100% higher counts of essential medicine compared to primary health centers. Given the aforementioned finding that better management may be associated with better outcomes, such as essential drug supply, these disparities may exacerbate inequities in health services and outcomes. Particularly for underserved populations in rural and lower-income communities, health facilities with better management may be inaccessible to those without the means to afford care at these places.

Based on these insights, policymakers may be able to target investments towards improving management in poorly performing facilities, so as to strengthen systems and downstream outcomes for the future. In so doing, they may be able to improve PHC service delivery and quality, particularly for disadvantaged populations.

This study has several strengths and limitations. A major strength is that this is one of the first studies in sub-Saharan Africa that assesses a nationally representative sample of both men and women over the age of 15 and their patterns of care-seeking and experience of care. In addition to surveying both women and men, the analysis also includes people of older ages who tend to be underrepresented in studies in sub-Saharan Africa. In terms of limitations, this is a cross-sectional study, so no causal relationships or directionality amongst variables can be determined. Also, though we controlled for several potential confounding variables, residual confounding may have contributed to our findings. Though we were able to assess important patient experience and facility process outcomes, our data and the respondents available through the PMA survey platform were limited, so we were unable to measure technical quality. Lastly, like many surveys, our assessments were open to selection bias (high performing facilities may have more responsive heads of facilities and thus be more likely to be represented in the study) and response biases such as social desirability bias and recall bias. We anticipate that the multifaceted nature of the PRIME-Tool likely mitigated the ability of a single measure to misrepresent the overall performance of the facility.

## Conclusions

Our study may be useful for Ugandan policymakers seeking to improve the readiness of the country’s health system, particularly following a significant stress to health facilities and supply chains like the COVID-19 pandemic. Through our findings and utilizing the management index, policymakers may be able to identify disparities across the Ugandan PHC system and reduce variability in the quality of facility management. Such interventions could have significant downstream effects such as better stocking of essential medicines and potentially the quality of care and health outcomes in Uganda.

## Data Availability

The data underlying the study is third party data owned by the Performance Monitoring for Action platform. The datasets supporting the conclusions of this article are available in the Performance Monitoring for Action repository at https://www.pmadata.org/data/available-datasets/request-access-datasets.

## Abbreviations

LMICs: Low and middle-income countries
PHC: Primary health care
PMA: Performance Monitoring for Action
PRIME-Tool: PRImary care facility Management Evaluation Tool
